# Associations of neighborhood characteristics with active park use: an observational study in two cities in the USA and Belgium

**DOI:** 10.1186/1476-072X-12-26

**Published:** 2013-05-07

**Authors:** Delfien Van Dyck, James F Sallis, Greet Cardon, Benedicte Deforche, Marc A Adams, Carrie Geremia, Ilse De Bourdeaudhuij

**Affiliations:** 1 Research Foundation Flanders (FWO), Egmontstraat 5, 1000, Brussels, Belgium; 2 Department of Movement and Sports Sciences, Ghent University, Watersportlaan 2, 9000, Ghent, Belgium; 3 Department of Family and Preventive Medicine, University of California San Diego, 3900 5th Avenue, Suite 310, San Diego, CA 92103, USA; 4 Department of Human Biometrics and Biomechanics, Vrije Universiteit Brussels, Pleinstraat 2, 1050, Brussels, Belgium; 5 School of Nutrition and Health Promotion, Arizona State University, 500 N Third Street, Phoenix, AZ 85004, USA

**Keywords:** Walkability, Income, SOPARC, EAPRS, Physical activity, Green space

## Abstract

**Background:**

Public parks can be an important setting for physical activity promotion, but to increase park use and the activity levels of park users, the crucial attributes related to active park use need to be defined. Not only user characteristics and structural park attributes, but also characteristics of the surrounding neighborhood are important to examine. Furthermore, internationally comparable studies are needed, to find out if similar intervention strategies might be effective worldwide. The main aim of this study was to examine whether the overall number of park visitors and their activity levels depend on study site, neighborhood walkability and neighborhood income.

**Methods:**

Data were collected in 20 parks in Ghent, Belgium and San Diego, USA. Two trained observers systematically coded park characteristics using the Environmental Assessment of Public Recreation Spaces (EAPRS) tool, and park user characteristics using the System for Observing Play and recreation in Communities (SOPARC) tool. Multilevel multiple regression models were conducted in MLwiN 2.25.

**Results:**

In San Diego parks, activity levels of park visitors and number of vigorously active visitors were higher than in Ghent, while the number of visitors walking and the overall number of park visitors were lower. Neighborhood walkability was positively associated with the overall number of visitors, the number of visitors walking, number of sedentary visitors and mean activity levels of visitors. Neighborhood income was positively associated with the overall number of visitors, but negatively with the number of visitors being vigorously active.

**Conclusions:**

Neighborhood characteristics are important to explain park use. Neighborhood walkability-related attributes should be taken into account when promoting the use of existing parks or creating new parks. Because no strong differences were found between parks in high- and low-income neighborhoods, it seems that promoting park use might be a promising strategy to increase physical activity in low-income populations, known to be at higher risk for overweight and obesity.

## Background

Lack of regular physical activity and prolonged sedentary time have independently been associated with numerous physical and mental health risks in all age groups (children, adolescents, adults and older adults) [1]. Nonetheless, large proportions of populations in developed countries do not engage in sufficient physical activity to gain health benefits [2]. Consequently, interventions to promote healthy and active lifestyles need to be improved and implemented. Ecological models of health behaviors emphasize the importance of taking into account multiple levels of influence when developing interventions. In addition to individual and social attributes, built environment and policy factors are expected to affect physical activity in multiple domains (e.g. leisure or transport) and settings (e.g. neighborhoods, parks) [3].

Public parks can be an important setting for physical activity promotion. Public parks are among the most common places for physical activity [4]. Parks are available in most communities, are usually free to access, can serve diverse populations including low-income and minority groups, and their provision can be influenced by local policies [5]. Thus, enhancing access to parks and optimizing their design seem to be promising strategies to increase physical activity.

To increase park use and the activity levels of park users, the crucial attributes related to active park use need to be defined. According to the conceptual model of Bedimo-Rung and colleagues [6] it is important to understand the associations of park user characteristics and structural park characteristics with visitation and physical activity within parks. Studies in the USA showed that park users were predominantly male and that adults and children were more likely to visit parks than adolescents and seniors [7-9]. Males and children were more likely to be active in parks than respectively, females and adults [7,8]. Furthermore, lower-income families were less likely to visit parks, while the evidence on racial/ethnic minorities was inconsistent [8-10]. Concerning the associations between specific structural park characteristics and park use, studies conducted in Australia and the USA showed that park availability, park size, and the availability and quality of amenities in parks were related with physical activity in parks among children, adolescents and adults [11-13].

In addition to examining park user and structural characteristics, it is necessary to examine neighborhoods and built environments where parks are located [6,14]. Since individuals must travel through the surrounding neighborhood before entering a park, neighborhood characteristics are likely to have an influence on whether and how a park is used [6]. Some neighborhood characteristics have been examined in previous research: low traffic safety, negative neighborhood aesthetics (e.g. abandoned housing) and low crime safety have been related to less park use [15-17]. Another potentially important neighborhood characteristic that has not been examined is neighborhood walkability (index including residential density, land use mix and street connectivity). High neighborhood walkability has been consistently related with physical activity in adults [18-20], but little is known about the availability and use of parks in high- and low-walkable neighborhoods. Possibly, parks are more prevalent, of higher quality and more easily accessible in high-walkable neighborhoods. Consequently, the overall higher amount of physical activity among high-walkable neighborhood inhabitants might be partially due to more frequent and active park use.

Neighborhood income is also an important environmental factor to take into account. Environmental characteristics like aesthetics, traffic safety infrastructure, crime safety and access to recreation facilities are less favorable in low-income neighborhoods [21,22], creating barriers to physical activity. It is not known whether park quality or physical activity in parks differ by neighborhood income.

Almost all studies examining the correlates of park use have been conducted in the USA or Australia. Because European built environments differ from American environments, it is important to conduct comparable studies across continents to determine whether similar study methods are applicable to different regions, if park use is comparable across regions and if similar factors are related with park use.

The present study addressed some of the shortcomings in the literature. The first aim was to describe structural park characteristics and socio-demographic characteristics of park visitors, and to examine if these attributes differed between Ghent, Belgium and San Diego, California, USA, between high- and low-walkable neighborhoods and between high- and low-income neighborhoods. Data of these two cities were used because of the need to compare novel European evidence with results of regions with more evidence on correlates of park use. As a second aim, we examined if study site, neighborhood walkability and neighborhood income were associated with the overall number of park visitors and their activity levels in parks.

## Methods

### Procedure

Data were collected in Ghent (Belgium) and San Diego, California (USA). Ghent has approximately 250,000 inhabitants, an area of 156.18 km^2^ and a population density of 1565 inhabitants/km^2^. Of all inhabitants, 18.8% belongs to ethnic-cultural minorities (mainly Turkish, Bulgarian and Moroccan); the other 81.2% is predominantly White. The distribution of inhabitants across age groups is as follows: 11.4% of the population is between 0 and 9 years, 9.9% between 10 and 19 years, 56.7% between 20 and 59, and 22.0% is older than 60 years of age (Belgian National Institute of Statistics 2011: http://www.statbel.fgov.be). San Diego counts 1,307,402 inhabitants, an area of 842.23 km^2^ and a population density of 1552 inhabitants/km^2^. San Diego’s ethnic racial distribution includes: 28.8% Hispanic/Latino, 26.2% other ethnic-racial minority (mainly Asian and African American); the other 45.0% is predominantly White. The age group distribution is as follows: 11.9% of the population is between 0 and 9 years, 12.7% between 10 and 19 years, 60.2% between 20 and 59, and 15.3% is older than 60 years of age (U.S. Bureau of the Census. American FactFinder, 2010 http://www.census.gov).

In both cities, a similar protocol was used. Parks were randomly selected from four quadrants categorized by crossing high/low walkability and high/low socioeconomic status of neighborhoods: high-walkable/high-income, high-walkable/low-income, low-walkable/high-income and low-walkable/low-income neighborhoods. These neighborhoods had been defined for previous studies examining the associations between the built environment and physical activity (Ghent; Belgian Environmental Physical Activity Study) [20] (San Diego; Neighborhood Impact on Kids study) [23]. The neighborhoods were chosen to maximize within-country variability in walkability and income. In both cities, neighborhoods consisted of clusters of administrative or population collection units (statistical sectors in Belgium, block groups in the USA), which were the smallest geographical units that had information on household income, other sociodemographic factors and objective spatial data for walkability.

In both cities, neighborhood-level walkability was determined objectively, using Geographic Information Systems (GIS)-based built environment parameters. In Ghent, neighborhood walkability included three environmental attributes previously found to be related to physical activity: net residential density, land use mix, and intersection density [24]. A detailed description of the calculation of this walkability index can be found elsewhere [24]. In San Diego, the neighborhood walkability index consisted of the same three variables plus retail floor area ratio [25]. In both cities, neighborhood-level income was determined using census-based median annual household income data (US census 2000: http://www.census.gov; Belgian National Institute of Statistics 2007: http://www.statbel.fgov.be).

In Ghent, 10 parks were randomly selected from a sampling frame of all parks in the four neighborhood quadrants: four parks were located in the low-walkable/low-income neighborhoods, while there were two parks in each of the other three quadrants. Afterwards, 10 parks were selected in San Diego after matching park size to the parks in Ghent. In San Diego, two parks were located in each of the high-walkable/low-income and the low-walkable/low-income neighborhoods, and three parks were located in each of the other quadrants. Information about park size was obtained from the City Council in Ghent and from GIS data provided in city records in San Diego, completed with data from Google Earth and information found on parks and recreation websites when needed.

In these 20 parks, park characteristics were systematically coded by two trained observers using the Environmental Assessment of Public Recreation Spaces (EAPRS) tool [14]. Characteristics of park users were observed using the System for Observing Play and Recreation in Communities (SOPARC) tool [8]. In Ghent, data were collected in August and September 2011 (summer season; mean temperature = 16.8°C, average number of days with precipitation = 10). In San Diego, SOPARC data were collected in October and November 2011 (mean temperature = 21.6°C, average number of days with precipitation = 3). EAPRS data had been collected previously by trained observers, from February to June 2008. Since 2008, no substantial park renovations were completed in the 10 selected parks in San Diego.

Two trained observers collected SOPARC and EAPRS data in Ghent and SOPARC data in San Diego. Before collecting SOPARC data, both observers completed SOPARC training, provided by Dr. McKenzie (on DVD). Before collecting EAPRS data in Ghent, the observers received a standard EAPRS training offered by the San Diego team who used the tool to collect the data in San Diego in 2008. In both cities, the first two parks were observed by both observers to ensure comparability and solve any inconsistencies between observers. The other eight parks were rated by one of the observers (each observer rated four parks per country).

In Ghent, the study was approved by the Ethics Committee of the Ghent University Hospital. In San Diego, the study was approved by the San Diego State University Institutional Review Boards.

### Measures

#### SOPARC

SOPARC is an objective observation tool to quantify the physical activity levels and socio-demographic characteristics of park users. SOPARC is a valid and reliable observation tool [8]. Use of SOPARC consists of defining discrete park zones, scanning a particular park zone, counting the overall number of park users in that zone, and classifying users by gender (males and females), age group (children, adolescents, adults, older adults), ethnicity (Latinos, Blacks, Non-Hispanic Whites, Other race/ethnicity) and physical activity level (sedentary, walking, vigorously active). Each park observation period was also classified by weather conditions (clear, cloudy, rainy) and darkness (dark, not dark). The number of park zones ranged from two to nine, and zones included open spaces, trails, playgrounds, swimming pools, basketball courts, sports fields, tennis courts, paths, picnic areas or shelters. In each park, observations were done during three days (two weekdays, one weekend day). On each observation day, four observation periods were conducted in each zone for about 15 minutes: in the morning (8AM), at noon, in the afternoon (3PM) and evening (7PM).

For the analyses, METs/observation period was calculated to obtain a representation of the average physical activity intensity during each observation period in a particular zone, independent of the number of visitors. To do so, a weighted MET score was given to each activity category (sedentary = 1 MET, walking = 3 METs, vigorous activity = 6 METs). These weighted MET scores were multiplied by the observed number of visitors in each physical activity category at the moment of the observation. Then, this score was divided by the total number of visitors present at the moment of the observation. This calculation produced a mean physical activity intensity score, independent of the number of visitors.

#### EAPRS

EAPRS is a detailed observation tool to assess park characteristics. The tool describes the physical environment of a park and focuses on the presence of park features and park amenities. Park features represent park characteristics that are essential to do physical activity. The observed park features included trails, paths, open spaces, swimming pools, playgrounds, sports fields and skating areas. Park amenities are aspects that contribute to the attractiveness of a park. The observed park amenities included places to sit, ponds/lakes, drinking fountains, picnic areas, vending amenities, restrooms, tables, bike racks and parking lots. EAPRS has good inter-rater reliability [14]. EAPRS was only used to obtain descriptive information about park characteristics.

### Statistical analyses

Descriptive statistics were analyzed using IBM SPSS Statistics 19. *χ*^2^ tests were conducted to examine associations of study site, neighborhood walkability and neighborhood income with gender, age, ethnicity and activity levels of the observed park visitors. One-way ANOVA tests were conducted to examine potential differences in park size, park features and park amenities between Ghent and San Diego, between high- and low-walkable neighborhoods, and between high- and low-income neighborhoods.

Multilevel multiple regression models were conducted in MLwiN 2.25 to examine the associations of the independent variables (study site, neighborhood walkability, neighborhood income) with the outcome measures (overall number of park visitors, number of visitors being sedentary, number of visitors walking, number of visitors being vigorously active, METs/observation period), after adjusting for covariates. Multilevel modeling was applied because the null-models showed that 4.4% to 8.1% of the variance in the outcome measures was attributable to differences between parks. Two levels were included in the analyses: observations (level 1 = individual level) and parks (level 2 = group level). For the analyses with number of park visitors, number of sedentary, walking and vigorously active visitors as the outcome, the Poisson distribution of the outcome measures was taken into account. For the analyses with METs/observation as an outcome measure, the skewed outcome measure was logarithmically transformed (log10) to improve its normality [26]. Park size, day type (weekday, weekend day), time of day (morning, noon, afternoon, evening), darkness (dark, not dark) and weather (cloudy, clear rainy) were included as covariates in all analyses. In the analyses with number of visitors sedentary, walking and vigorously active as outcomes, the total number of visitors per observation was included as an additional covariate. For all analyses, significance was set at p < 0.05.

## Results

### Associations of study site, walkability and income with socio-demographic characteristics of park users and park characteristics

Descriptive characteristics of the parks and park users by study site, neighborhood walkability and neighborhood income are shown in Table 1. Results of the *χ*^2^ tests showed that all associations of site, neighborhood walkability and neighborhood income with gender, age group, ethnicity and activity level of the park visitors were significant (all p < 0.034).

**Table 1:** Descriptive characteristics of parks and park users by study site, neighborhood walkability and neighborhood income

		**Study site**		**Walkability**		**Income**	
	**Total**	**Ghent**	**San Diego**	**Low**	**High**	**Low**	**High**
**Number of parks**	20	10	10	11	9	11	9
**Park size in hectare** (mean [SD])	7.6 (7.7)	6.0 (5.8)	8.5 (8.4)	10.5 (8.6)	3.2 (1.5)	4.4 (4.3)	10.7 (8.8)
**SOPARC observations**							
*Total observed individuals* (n)	1836	766	1070	958	878	699	1137
*Gender* (n[%])							
Male	1099 (59.9)	393 (51.3)	706 (66.0)	622 (64.9)	477 (54.3)	440 (62.9)	659 (58.0)
Female	737 (40.1)	373 (48.7)	364 (34.0)	336 (35.1)	401 (45.7)	259 (37.1)	478 (42.0)
*Age group* (n[%])							
Children	409 (22.3)	106 (13.8)	303 (28.3)	212 (22.1)	197 (22.4)	94 (13.4)	315 (27.7)
Adolescents	509 (27.7)	350 (45.7)	159 (14.9)	225 (23.5)	284 (32.3)	122 (17.5)	387 (34.0)
Adults	861 (46.9)	270 (35.3)	591 (55.2)	491 (51.3)	370 (42.0)	438 (62.7)	423 (37.2)
Older adults	57 (3.1)	40 (5.2)	17 (1.6)	30 (3.1)	27 (0.3)	45 (6.4)	12 (1.1)
*Ethnicity* (n[%])							
Latino	205 (11.2)	0 (0)	205 (19.2)	135 (14.1)	70 (7.9)	111 (15.9)	94 (8.3)
Black	138 (7.5)	5 (0.7)	133 (12.4)	48 (5.0)	90 (10.3)	78 (11.2)	60 (5.3)
White	1238 (67.4)	685 (89.4)	553 (51.7)	657 (68.6)	581 (66.2)	434 (62.1)	804 (70.7)
Other	249 (13.6)	70 (9.1)	179 (16.7)	118 (12.3)	131 (14.9)	72 (10.3)	177 (15.6)
Missing	6 (0.3)	6 (0.8)	0 (0)	0 (0)	6 (0.7)	4 (0.5)	2 (0.1)
*Activity level* (n[%])							
Sedentary	825 (44.9)	410 (53.5)	415 (38.8)	344 (35.9)	481 (54.8)	283 (40.5)	542 (47.7)
Walking	336 (18.3)	159 (20.8)	177 (16.5)	160 (16.7)	176 (20.0)	140 (20.0)	196 (17.2)
Vigorous activity	663 (36.1)	185 (24.2)	478 (44.7)	454 (47.4)	209 (23.8)	273 (39.1)	390 (34.3)
Missing	12 (0.7)	12 (1.5)	0 (0)	0 (0)	12 (1.4)	3 (0.4)	9 (0.8)
*METs/observation* (mean [SD])	1.1 (1.8)	1.2 (1.8)	1.0 (1.9)	1.1 (1.9)	1.1 (1.7)	1.1 (1.9)	1.0 (1.8)
**EAPRS observations**							
*% of parks with park features*							
Trail	40.0	40.0	40.0	45.5	33.3	54.5	22.2
Path	100.0	100.0	100.0	100.0	100.0	100.0	100.0
Open space	80.0	90.0	70.0	90.9	66.7	81.8	77.8
Swimming pool	5.0	0.0	10.0	9.1	0.0	0.0	11.1
Play area	85.0	80.0	90.0	100.0	66.7	82.8	88.9
Sports field	55.0	40.0	70.0	63.6	44.4	45.5	66.7
Skating area	0.0	0.0	0.0	0.0	0.0	0.0	0.0
*% of parks with park amenities*							
Places to sit	90.0	90.0	90.0	100.0	77.8	90.9	88.9
Pond/lake	0.0	0.0	0.0	0.0	0.0	0.0	0.0
Drinking fountain	40.0	10.0	70.0	45.5	33.3	45.5	33.3
Picnic area	50.0	0.0	100.0	45.5	55.6	0.0	55.6
Vending amenities	15.0	10.0	20.0	18.2	11.1	54.5	33.3
Restroom	60.0	30.0	90.0	63.6	55.6	54.5	66.7
Table	50.0	10.0	90.0	36.4	66.7	45.5	55.6
Bike racks	50.0	40.0	60.0	45.5	55.6	36.4	66.7
Parking lot	50.0	30.0	70.0	45.5	55.6	18.2	88.9
*Total park features* (mean [SD])	3.8 (0.8)	3.5 (0.7)	3.8 (1.4)	4.1 (0.5)	3.1 (1.1)	3.6 (1.1)	3.7 (0.7)
*Total park amenities* (mean [SD])	4.7 (2.1)	2.2 (1.5)	5.9 (1.1)	4.0 (2.4)	4.1 (2.3)	3.4 (2.0)	4.9 (2.4)

SD = standard deviation.

Concerning gender, male visitors were more prevalent in San Diego, low-walkable neighborhood parks and low-income neighborhood parks than in Ghent, high-walkable neighborhood parks and high-income neighborhood parks, respectively.

Regarding the age group of the visitors, children and adults were more prevalent in San Diego than in Ghent, while adolescents and older adults were more prevalent in Ghent. In high-walkable neighborhood parks, more adolescents, fewer adults and fewer older adults were observed than in low-walkable neighborhoods. In the high-income neighborhood parks more children, adolescents, fewer adults and fewer older adults were observed than in the low-income neighborhoods.

Concerning the ethnicity of the park visitors, a mix of different ethnicities was observed in San Diego parks, while in Ghent, 89.4% of the visitors were Non-Hispanic White. In low-walkable neighborhood parks, more Latinos and fewer Blacks were observed than in high-walkable neighborhoods. In low-income neighborhoods Latinos and Blacks were more prevalent than in high-income neighborhood parks, while Whites and people from other ethnicities were less prevalent.

Regarding the activity level of the park visitors, sedentary people were more prevalent in Ghent, while vigorously active people were more prevalent in San Diego. In high-walkable neighborhood parks, more sedentary visitors and fewer vigorously active visitors were observed than in low-walkable neighborhoods. In low-income neighborhoods, fewer sedentary visitors and more vigorously active visitors were observed than in high-income neighborhood parks.

The one-way ANOVA tests showed that mean park size was higher in low-walkable and high-income neighborhoods (both p < 0.001). Mean number of park features was higher in San Diego and low-walkable neighborhoods than in Ghent and high-walkable neighborhoods (both p < 0.001). No difference in park features was found between high- and low-income neighborhoods (p = 0.703). Mean number of park amenities was higher in San Diego and in high-income neighborhoods (both p < 0.001), but did not differ significantly between high- and low-walkable neighborhoods (p = 0.065).

### Independent associations of covariates with outcome measures

Results of the two-level multiple regression analyses are shown in Table 2. Park size was negatively related to the number of sedentary visitors (p < 0.001), and positively to the number of vigorously active visitors (p < 0.05). On weekdays, the overall number of park visitors was higher than on weekend days (p < 0.001), but the number of vigorously active visitors was higher on weekend days (p < 05). Compared with the observations in the morning, more visitors (overall) and more sedentary visitors were observed at noon, in the afternoon and in the evening (all p < 0.001). However, fewer visitors were observed walking at noon (p < 0.001), in the afternoon (p < 0.001) and in the evening (p < 0.01), when compared with the morning observations. In the afternoon, more vigorously active visitors were observed (p < 0.05), and METs/observation were higher at noon (p < 0.01) and in the afternoon (p < 0.001) than in the morning. Compared with observations when it was dark, the number of sedentary visitors and number of visitors walking were higher during daylight (both p < 0.05). Finally, compared with observations during cloudy weather, the number of sedentary visitors and METs/observation were higher when the weather was clear (both p < 0.05). The total number of visitors was lower when the weather was rainy (p < 0.001), but during rainy weather, more vigorously active visitors were observed than in cloudy weather (p < 0.05).

**Table 2:** Associations of covariates, neighborhood walkability, income and study site with the different outcome measures: results of two-level multiple regression analyses

	**Number of visitors per observation^a^**	**Number of visitors sedentary per observation^b^**	**Number of visitors walking per observation^b^**	**Number of visitors doing vigorous PA per observation^b^**	**METs per observation^a^**
	**β (SE)**	**β (SE)**	**β (SE)**	**β (SE)**	**β (SE)**
**Covariates**					
Park size	0.011 (0.032)	−0.038 (0.009)***	−0.021 (0.026)	0.046 (0.021)*	0.077 (0.048)
Day type (ref = weekday)	−0.396 (0.054)***	−0.141 (0.093)	−0.185 (0.130)	0.186 (0.101)*	0.448 (0.752)
Time of day (ref = morning)					
Noon	1.321 (0.087)***	1.026 (0.185)***	−0.562 (0.164)***	−0.253 (0.172)	2.321 (0.996)**
Afternoon	1.657 (0.081)***	0.848 (0.183)***	−0.722 (0.156)***	0.314 (0.158)*	4.179 (0.993)***
Evening	0.449 (0.112)***	0.798 (0.216)***	−0.529 (0.209)**	−0.170 (0.193)	0.240 (1.150)
Darkness (ref = dark)	0.147 (0.126)	0.446 (0.246)*	0.604 (0.353)*	−0.095 (0.206)	1.880 (1.359)
Weather (ref = cloudy)					
Clear	0.080 (0.079)	0.242 (0.127)*	−0.078 (0.176)	−0.068 (0.153)	1.542 (0.811)*
Rainy	−1.031 (0.310)***	−1.418 (1.085)	−1.008 (1.056)	0.817 (0.427)*	−1.412 (2.643)
Number of visitors/observation		3.160 (0.082)***	3.140 (0.122)***	3.596 (0.102)***	
**Main effects**					
Study site (ref = Ghent)	−0.373 (0.048)***	−0.020 (0.083)	−0.376 (0.119)***	0.552 (0.103)***	1.437 (0.743)*
Walkability (ref = low)	0.766 (0.059)***	0.284 (0.091)***	0.653 (0.147)***	−0.152 (0.116)	2.096 (0.814)***
Income (ref = low)	0.212 (0.055)***	0.031 (0.090)	−0.062 (0.136)	−0.765 (0.108)***	−0.122 (0.806)

PA = physical activity.

* p < 0.05; ** p < 0.01; *** p < 0.001.

*Note:* Park size is a level-2 variable; all other covariates are level-1 variables.

^a^ regression analyses adjusted for park size, day type, time of day, darkness and weather.

^b^ regression analyses adjusted for park size, day type, time of day, darkness, weather and number of visitors per observation.

### Associations of neighborhood walkability, income and study site with outcome measures

Results of the regression analyses are presented in Table 2. In San Diego, METs/observation were higher (p < 0.05), as well as the number of vigorously active visitors (p < 0.001). In Ghent, the overall number of visitors per observation and the number of visitors walking were higher (both p < 0.001). Neighborhood walkability was positively associated with the overall number of visitors per observation, with the number of visitors walking, the number of visitors being sedentary and with METs/observation (all p < 0.001). Neighborhood income was positively associated with the overall number of visitors per observation (p < 0.001) but negatively with the number of visitors being vigorously active (p < 0.001).

## Discussion

Overall, park users were more likely to be male, and Non-Hispanic White, than female or Black, Latino or of another ethnicity. These results are in agreement with previous findings showing that men are more likely to visit parks than women [8,9]. The higher prevalence of Non-Hispanic Whites seems to be a reflection the overall composition of the populations in Ghent and San Diego. Previous results also showed that adults and children are more likely to visit parks than adolescents and older adults [8,9]: this was the case in San Diego, but in Ghent, mainly adults and adolescents visited the parks. A large proportion (44.9%) of the park users was observed being sedentary. This is an important observation, pointing out that park visits do not necessarily induce or stimulate active park use.

This study revealed large differences in park and user characteristics across the two study sites. When interpreting these differences, it should be taken into account that the mean temperature during data collection was higher in San Diego than in Ghent, possibly affecting the findings. The regression analyses showed that after taking into account the covariates, park users in San Diego were more likely to be vigorously active, and the average intensity level of activities (defined as METs/observation) was higher than in Ghent. On the other hand, the total number of park visitors was higher in Ghent, as well as the number of visitors walking. The greater number of vigorously active park visitors in San Diego might be explained by more activity-supportive park features in San Diego parks than in parks in Ghent. For example, swimming pools, play areas and sports field were more prevalent in San Diego than in Ghent. Additionally, the higher activity-related intensity level of park users in San Diego could possibly be explained by the fact that USA cities usually are more car-oriented than European cities [27]. In San Diego, parks probably play a more significant role as environments to be physically active, rather than in Ghent, where many people are active on the safer streets for both leisure and transport purposes (e.g. jogging, cycling). In Ghent, parks are possibly perceived more as a place to relax or walk instead of a place to be vigorously active.

After taking into account the covariates (e.g. park size, darkness), the activity-related intensity level (METs/observation), total number of visitors, and number of visitors sedentary and walking were higher in high-walkable neighborhoods than in low-walkable neighborhoods. Previous studies showed that living in a high-walkable neighborhood was associated with more active transportation in adults [18-20] and the present findings show that neighborhood walkability also contributes to more (active) park use. Perhaps easier access to parks in high-walkable neighborhoods facilitates more visits to both sedentary and walking pursuits. Higher walkability may be related to more visitors walking to parks from their homes. Until now, no studies examined neighborhood walkability as a correlate of park use, but other environmental characteristics like high traffic safety, high crime safety and high aesthetics have been associated with higher park use [15-17]. These features are usually more favorable in high-walkable neighborhoods [28,29] so present results confirm these previous findings.

The higher number of sedentary park users in high-walkable neighborhood parks might be explained by the fact that fewer park features were present in these parks. Furthermore, results from a previous study conducted in Ghent showed that high neighborhood walkability was not only related to more physical activity, but also (and independently) to more sedentary time [30]. Similarly, high neighborhood walkability might stimulate active (i.e. walking), as well as sedentary park use. Possibly, high-walkable neighborhood characteristics stimulate inhabitants to go outside, not only to be active but also to be sedentary in parks.

After controlling for the covariates, neighborhood income was positively related to the total number of observed visitors, but negatively to the number of vigorously active visitors. Interestingly, the average intensity level of park users did not differ between high- and low-income neighborhood parks, neither did the number of activity-supportive park features. This is encouraging, as low-income individuals are often found to be at higher risk of insufficient physical activity and overweight/obesity [31]. It seems that in Ghent and San Diego, both parks in high- and low-income neighborhoods have features (e.g. open spaces, play areas, sports fields) that can encourage physical activity, and parks are a promising environment to stimulate (vigorous) physical activity. Parks in high-income neighborhoods had more park amenities, possibly leading to increased park attractiveness and a higher overall number of park visitors. Creating parks and improving the quality (features and amenities) of available parks might be an effective approach to reach population subgroups at risk for overweight and obesity.

In accordance with the conceptual model of Bedimo-Rung and colleagues [6], not only user and park characteristics, but also characteristics of the surrounding neighborhood are important to understand park use. Nonetheless, the present study could not confirm if park users live in the neighborhoods surrounding the parks they use or not. This remains to be explored in further studies, possibly by including interviews with park users. It would also be useful to collect information on the transportation modes used to visit parks. It might be that individuals are more likely to use active transportation modes to visit parks that are located in high-walkable neighborhoods compared with parks in low-walkable neighborhoods. This can be important to fully understand the role parks play to stimulate physical activity. Moreover, future studies should examine how active park use can be encouraged in low-walkable neighborhoods, because the quality (i.e. park features and amenities) of parks in the low-walkable neighborhoods was generally high, but (active) park use was still lower than in the high-walkable neighborhoods.

The present study has several strengths. First, observational data, measured with valid and reliable observation tools, were used to assess park use and park characteristics. Second, an identical study protocol was used in Ghent and San Diego, making it possible to compare the findings across two cities in different countries. These comparisons revealed large and surprising differences. Third, the same trained observers collected SOPARC data in Ghent and San Diego. For EAPRS data collection, the observers received a standard training offered by the team who collected the data in San Diego. Applying these procedures increased the comparability of data between cities. However, study limitations also need to be acknowledged. First, no inter-rater reliability data were collected and although all observers were trained and certified during the training, some discrepancies in observations between different raters may still have been present. Second, because of the limited number of parks (n = 20), the associations between park features/amenities and park use could not be analyzed statistically in the present study. Third, because of practical limitations, SOPARC data were only collected over three days, while Cohen and colleagues [32] recommend four days of data collection. Fourth, no background information of park users was collected, making it impossible to know if park users lived in the neighborhood around the parks or not.

In conclusion, the present study showed that in addition to park and user characteristics, neighborhood characteristics are important to explain park use. The results showed that the presence of parks did not necessarily induce active park use; a large proportion of the park users were observed being sedentary. Furthermore, (active) park use was higher in San Diego and in high-walkable neighborhood parks. The overall number of park visitors was lower in low-income neighborhoods, but interestingly, visitors of low-income neighborhood parks were more vigorously active and the overall activity-related intensity did not differ between high- and low-income neighborhoods. So, it seems that the promotion of active use of existing parks or creating new parks can be a promising strategy to increase physical activity in population subgroups at risk for overweight and obesity.

## Competing interests

The authors declare that they have no competing interests.

## Authors’ contributions

All authors contributed to the design of different parts of the study. DVD coordinated the Belgian data collection, conducted the statistical analyses and drafted the manuscript. CG coordinated the USA data collection. GC, IDB, JFS, MAA, BD and CG participated in the interpretation of the data, helped to draft the manuscript and revised the manuscript for important intellectual content. All authors read and approved the final manuscript.
